# Bromelain-Infused Poly(vinyl alcohol)/Hydroxyethyl Cellulose Nanofibrous Scaffolds for Cancer Therapy: Fabrication, Characterization, and *In Vitro* Assessment

**DOI:** 10.1021/acsomega.4c09347

**Published:** 2025-05-15

**Authors:** Suganya Bharathi Balakrishnan, Lilian Ibrahim, Esakkimuthu Shanmugasundaram, Na’il Saleh, Stalin Thambusamy

**Affiliations:** † Department of Chemistry, School of Engineering, 160278 Dayananda Sagar University, Devarakaggalahalli, Harohalli, Ramanagara 560 078, Karnataka, India; ‡ Department of Chemistry, College of Science, 11239 United Arab Emirates University, P.O. Box 15551, Al Ain 15551, United Arab Emirates; § Department of Industrial Chemistry, School of Chemical Sciences, Alagappa University, Karaikudi 630003, Tamil Nadu, India

## Abstract

Nanofibrous scaffolds based on biomaterials have recently received a lot of attention due to their unique physicochemical characteristics. In this article, we disclose the encapsulation of bromelain in a poly­(vinyl alcohol) (PVA)/hydroxyethyl cellulose (HEC) matrix to create a new type of nanofibrous scaffold. Bromelain has been used to impart new properties to PVA/HEC nanofibrous scaffolds, such as antibacterial, antioxidant, and biocompatibility. After examining the physicochemical properties of the nanofibrous scaffolds, it was discovered that bromelain was successfully incorporated into PVA/HEC nanofibers, providing a significant morphological structure to the scaffolds. The *in vitro* release study indicated that the loaded bromelain exhibited a sustained and controlled release behavior from the PVA/HEC nanofibrous scaffolds, thereby effectively inhibiting the growth of HeLa cells, as evidenced by microscopic observations. It should be emphasized that the PVA/HEC nanofibrous scaffold containing bromelain may have a promising application for cancer therapy.

## Graphical abstract

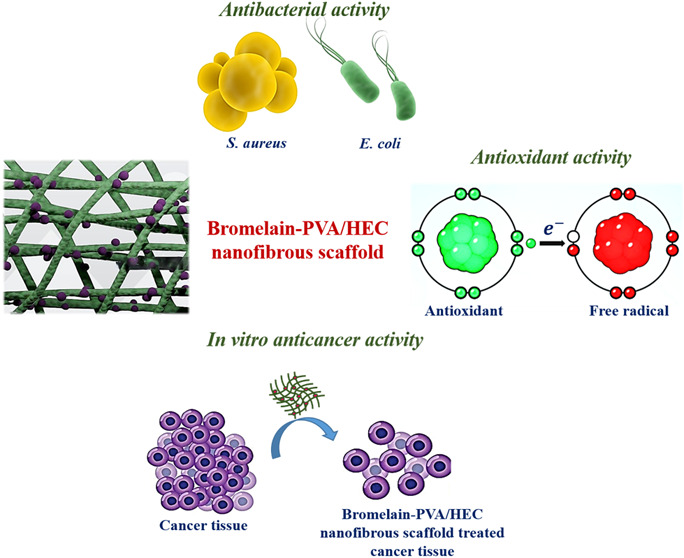

## 1 Introduction

Nanofibrous scaffolds, one of the most extensively explored biomaterials, are commonly used in cell and tissue responses.^1–3^ Cell toxicity studies are a useful initial step in determining the potential toxicity of a test substance, plant extracts, biologically active compounds, or nanoparticles.^4–6^ Cell culture, including cytotoxicity and cell viability assays, is the most important screening method currently used in life science research for medical device or biomaterial biocompatibility screening.^7,8^ It should be noted that the cytotoxicity assay is a preliminary and essential component of other *in vitro* toxicity studies, which are frequently used to identify the most promising molecules to be studied *in vivo*.^9,10^ The toxicity of biologically active compounds is largely attributed to their ability to generate oxygen free radicals, as well as some specific or nonspecific interactions with biological structures and biomacromolecules.^11^ In this regard, several researchers were reported to exhibit a strong, potent anticancer impact on cervical cancer HeLa cells. Therefore, a novel class of nanofibrous scaffolds is being created to enhance the cell viability profile of biomaterials.

With the development of electrospinning, the use of electrospun nanofibrous scaffolds as appropriate drug carriers in therapeutic delivery remains a great challenge.^12–16^ Polymer-based nanofibrous scaffolds have received significant attention in the biomedical field because of their functionality, biodegradability, biocompatibility, and high loading capacity for biological substances and active species.^14,17^ Scaffolds with a suitable surface-to-volume ratio, an interconnected geometry, and structural strength have been produced from various synthetic and natural polymers, including poly­(methyl methacrylate) (PMMA), polyvinylpyrrolidone (PVP), poly­(ethylene oxide) (PEO), polycaprolactone (PCL), poly­(lactic-*co*-glycolic acid) (PLGA), cellulose, lignin, chitosan, alginate, dextran, gelatin, and hyaluronic acid. Electrospinning the natural polymer itself proved difficult, due to its limited solubility and electrospinnability.^18–22^ To address the challenges associated with the electrospinnability of natural polymers, we created nanofibrous scaffolds from HEC and PVA.

HEC, a nonionic hydrophilic polysaccharide composed of glucose units linked together by β-glycosidic linkage, is widely used in biomedical devices, tissue engineering materials, and wound dressings.^23^ Electrospinning HEC alone was difficult due to the poor dispersion of several composites and its nonionic nature. However, to enhance the solubility and compatibility of HEC, PVA seems to be the most prevalent choice for the fabrication of conventional scaffolds because of its mechanical strength and flexibility.^24–26^ Takeno et al. synthesized PVA hydrogel films with cellulose nanofibers that were cross-linked using borax by the freezing method; the dual cross-linking resulted in a significant increase in mechanical properties.^27^ In terms of these, HEC cross-linked with PVA is an effective method for fabricating nanofibrous scaffolds with promising properties.

In recent years, numerous naturally occurring dietary compounds have demonstrated significant anticancer activity.^28–30^ Among them, bromelain, a sulfhydryl proteolytic enzyme isolated from pineapple fruit and stem, contains a mixture of different thiol endopeptidases and nonprotease components.^31,32^ Studies have demonstrated that bromelain possesses a wide range of therapeutic benefits, including antioxidant, anti-inflammatory, immunomodulatory, antithrombotic, cardioprotective, wound healing, and anticancer properties. Bromelain’s protease components are primarily responsible for its anticancer properties.^33–37^ pH-sensitive bromelain-based nanoparticles for effective drug delivery and tumor treatment were demonstrated by Tang et al.^38^ In several reports, bromelain is said to enhance resistance against the proliferation of cancerous cells.

We previously demonstrated the potential use of electrospun polymer nanofibrous scaffolds as carriers for effective wound healing. We hypothesized that the incorporation of enzymes into an electrospun PVA/HEC nanofibrous scaffold might offer significant benefits to cancer treatment. This work aims to investigate the anticancer potential of bromelain-infused PVA/HEC nanofibers as a proof of concept. Bromelain-loaded PVA/HEC nanofibrous scaffold was fabricated via an electrospinning approach. The physicochemical properties and biological characteristics of the nanofibrous scaffolds were examined. Thus, the fabricated nanofibrous scaffolds were investigated *in vitro* against malignant HeLa cells for potential cancer treatment.

## 2 Materials and Methods

### 2.1 Materials

Poly­(vinyl alcohol) (*M*_w_ = 10,000) was purchased from HiMedia Laboratories Pvt. Ltd. Hydroxyethyl cellulose (*M*_w_ = 800–1500 mPa·s, 2% in water at 20 °C) was obtained from TCI Chemicals (India) Pvt. Ltd. Bromelain and lysozyme (egg white (Muramidase) 15000 U/mg) were provided by Sisco Research Laboratories (SRL) Pvt. Ltd. All the chemicals received were of analytical grade and used without any further purification. Deionized water was used throughout the work.

### 2.2 Methods

#### 2.2.1 Nanofibrous Scaffolds Fabrication via Electrospinning Technique

The electrospinning solution was prepared by dissolving 10% (w/v) PVA and 5% (w/v) HEC in deionized water, and the mixture was heated at 80 °C and mixed using a thermal magnetic stirrer until all the PVA and HEC were completely dissolved.^23,25^ Then, the solution was left on a magnetic stirrer for 6 h. Briefly, 5% (with respect to PVA:HEC) bromelain was added to the PVA/HEC solution with continuous stirring to obtain a homogeneous solution. A certain amount of bromelain-PVA/HEC solution was withdrawn with a 10 mL syringe, respectively. The drum collector was placed 15 cm away from the syringe needle tip. The electrospinning process was carried out under ambient conditions with an output voltage of 16 kV and a feeding rate of 0.7 mL/h. The fabricated nanofibers were collected on the expansion cylinder rotating at 700 rpm, dried overnight at room temperature, and then used for further studies.

### 2.3 Material Characterizations

The electrospun nanofibrous scaffolds were fabricated in the laboratory by using electrospinning equipment (ESPIN-NANO) procured from Physics Instrument Company, Chennai. The surface morphology of the obtained electrospun nanofibrous scaffolds was viewed using scanning electron microscopy (SEM, FEI-Quanta 250 FEG). Direct electrospinning is used to deposit nanofibers on the Carbon-supported Copper-grid (mesh size −200), with voltage and distance optimized for homogeneous deposition, and the samples analyzed using high-resolution transmission electron microscopy (HR-TEM, JEOL 3010 at 300 kV). The diameters of the prepared nanofibers were analyzed from the SEM images using image processing software (ImageJ), with the average value calculated from 50 measurements. Atomic force microscopy (AFM) measurements (NT-MDT, Model TD150, Russia) were performed on both samples using a multimode scanning probe microscope. Attenuated total reflectance Fourier transform infrared (ATR-FTIR) spectroscopic analysis of the nanofibrous scaffolds was performed in transmittance mode with a Jasco 4600 Type A spectrometer over the range of 4000–550 cm^–1^ at a resolution of 4 cm. A UV–vis-NIR spectrophotometer (Jasco V-670 spectrophotometer) was used to record the absorbance spectra. The X-ray diffraction patterns of the prepared nanofibrous scaffold were measured for phase and crystallinity using an X’Pert PRO diffractometer. Thermal properties, weight loss, and thermal stability of the scaffolds were determined by differential scanning calorimetry (DSC) and thermogravimetric analysis (TGA) from 50 to 800 °C at a heating rate of 10 °C/min using the STA 409 PC/PG NETZCH instrument.

### 2.4 Porosity Measurement

The porosity of the prepared nanofibrous scaffolds was measured by the liquid displacement method.^18^ In this assay, the scaffolds were cut into 1 × 1 cm^2^ pieces and then immersed in absolute ethanol until they were saturated. The excess liquid on the surface of the scaffolds was removed by filter paper after taking out the ethanol. Subsequently, the samples were weighed, and the porosity of the scaffolds was evaluated by determining the amount of ethanol absorbed by the scaffolds using the following equation

$$P=\frac{W_{2}-W_{1}}{\rho V}\times 100$$

where *W*_1_ is the initial weight of the dry scaffolds and *W*_2_ is the weight of the swollen nanofibrous scaffolds, respectively. *V* is the volume of nanofibrous scaffolds before immersion in ethanol. ρ is the density of ethanol at room temperature (789 kg/m^3^). All samples were triplicated in the experiment.

### 2.5 Swelling Profile

The *in vitro* swelling behavior of the nanofibrous scaffolds was evaluated by cutting the scaffold into a square piece (1 × 1 cm^2^ size) and immersing them into phosphate buffered saline (PBS, pH ∼ 7.4) at 37 °C under continuous stirring.^20^ At a predetermined time interval, samples were retrieved from the buffer solution. The weights of the scaffolds were measured after removing the surface wetness by using filter paper and were hung for 1 min to get rid of excessive moisture. The equilibrium-swelling ratio was calculated using the following equation

$$\mathrm{swelling}\;\left(\%\right)=\frac{W_{f}-W_{i}}{W_{i}}\times 100$$

where *W*_i_ is the initial weight and *W*_f_ is the swelled weight of the scaffold, respectively.

### 2.6 *In Vitro* Enzymatic Degradation

The *in vitro* degradation study of the nanofibrous scaffolds was performed to measure the rate of degradation and their biological stability.^39^ Briefly, scaffolds of known dry weights (*W*_0_) were exposed to lysozyme enzyme (10,000 U/mL) for 21 days at 37 °C (pH ∼ 7.4). The test samples were removed after specific time intervals (7, 14, and 21 days) from the medium containing lysozyme and rinsed with deionized water. The excess solution was removed from the surface by using tissue paper and weighed (*W*_d_). The degradation rate was calculated using the following equation

$$\mathrm{degradation}\;\left(\%\right)=\frac{W_{0}-W_{d}}{W_{0}}\times 100$$

### 2.7 *In Vitro* Drug Release

The release of bromelain from the PVA/HEC nanofibrous scaffold was measured using a UV–vis instrument (Shimadzu UV-2401 spectrophotometer) at an optical wavelength of 265 nm.^20^ Samples of nanofibers (50 g) were placed in 50 mL of PBS (pH ∼ 7.4) and continuously shaken at room temperature. An aliquot sample was withdrawn at specific time intervals, and the same amount of fresh PBS was added to the release medium to maintain the sink condition. All samples were studied in triplicate.

### 2.8 Antibacterial Activity

The *in vitro* antibacterial activity of the nanofibrous scaffolds was tested against *Staphylococcus aureus* (*S. aureus*) and *Escherichia coli* (*E. coli*) by the disc agar diffusion method using Mueller-Hinton agar (MHA). Concisely, the bacterial strains were inoculated and cultured in MHA at 37 °C overnight and serially diluted to 1 × 10^8^ colony-forming units (CFU)/mL. The nanofibrous scaffolds (1 × 1 cm^2^ piece) were placed on the surface of the MHA. After contact with the electrospun mats, the inhibitory zone is measured and evaluated. All tests were done in triplicate.

### 2.9 Antioxidant Activity

The antioxidant activity of fabricated nanofibrous scaffolds was analyzed by a 2,2-diphenyl-1-picrylhydrazyl (DPPH) assay. The nanofibrous scaffolds with different concentrations (5, 25, 50, 75, and 100 μg/mL) were added to 4 mL of a 200 μM solution in methanol. The reaction mixture was then incubated in the dark for 1 h. Afterward, the absorbance was determined at 517 nm using a UV–vis spectrophotometer. All the samples were carried out in triplicate. DPPH scavenging activity was calculated using the formula

$$\mathrm{percentage}\;\mathrm{of}\;\mathrm{DPPH}\;\mathrm{scavenging}=\frac{A_{c}-A_{s}}{A_{c}}\times 100$$

where *A*_c_ is the absorbance of the control at 517 nm, and *A*_s_ is the absorbance of different nanofibrous samples at 517 nm. The results were expressed as IC_50_, which is the concentration of the nanofibrous scaffolds required to inhibit 50% of the DPPH concentration.

### 2.10 *In Vitro* Cytotoxicity Assay

A cervical cancer cell line (HeLa) was obtained from the National Centre for Cell Science (NCCS), Pune, and cultured in Eagle’s minimum essential medium (MEM) supplemented with 10% fetal bovine serum (FBS) and 1% antibiotic in an atmosphere of 5% CO_2_, 95% air, and 100% relative humidity at 37 °C. The monolayer cells were detached with trypsin-EDTA from the culture plate and were seeded on the nanofibrous scaffolds at a density of 1 × 10^5^ cells per well. The biocompatibility of the nanofibrous scaffolds was evaluated using the MTT assay (3-[4,5-dimethylthiazol-2-yl]-2,5-diphenyl tetrazolium bromide) at different culture periods up to 24 h in triplicate. MTT solution was added to each well and incubated for 4 h. The formazan complex was dissolved in dimethyl sulfoxide (DMSO), and the optical density was calculated with a microplate reader at a wavelength of 570 nm. The percentage of cell viability was calculated from the optical density of samples and controls.

### 2.11 Statistical Analysis

The data represents the mean ± standard deviation (SD) of three independent replication studies. Statistical significance was established using the *t* test in SPSS software, with *p*-values of less than 0.05 being statistically significant.

## 3 Results and Discussion

### 3.1 Morphology of Electrospun Nanofibrous Scaffolds

Figure 1. shows the surface morphology and diameter histograms of the neat PVA/HEC and bromelain-PVA/HEC nanofibrous scaffolds. The absence of a bead in the fiber structure resulted in smooth and uniform fibers (Figure 1(a),(b)).

**Figure 1:**
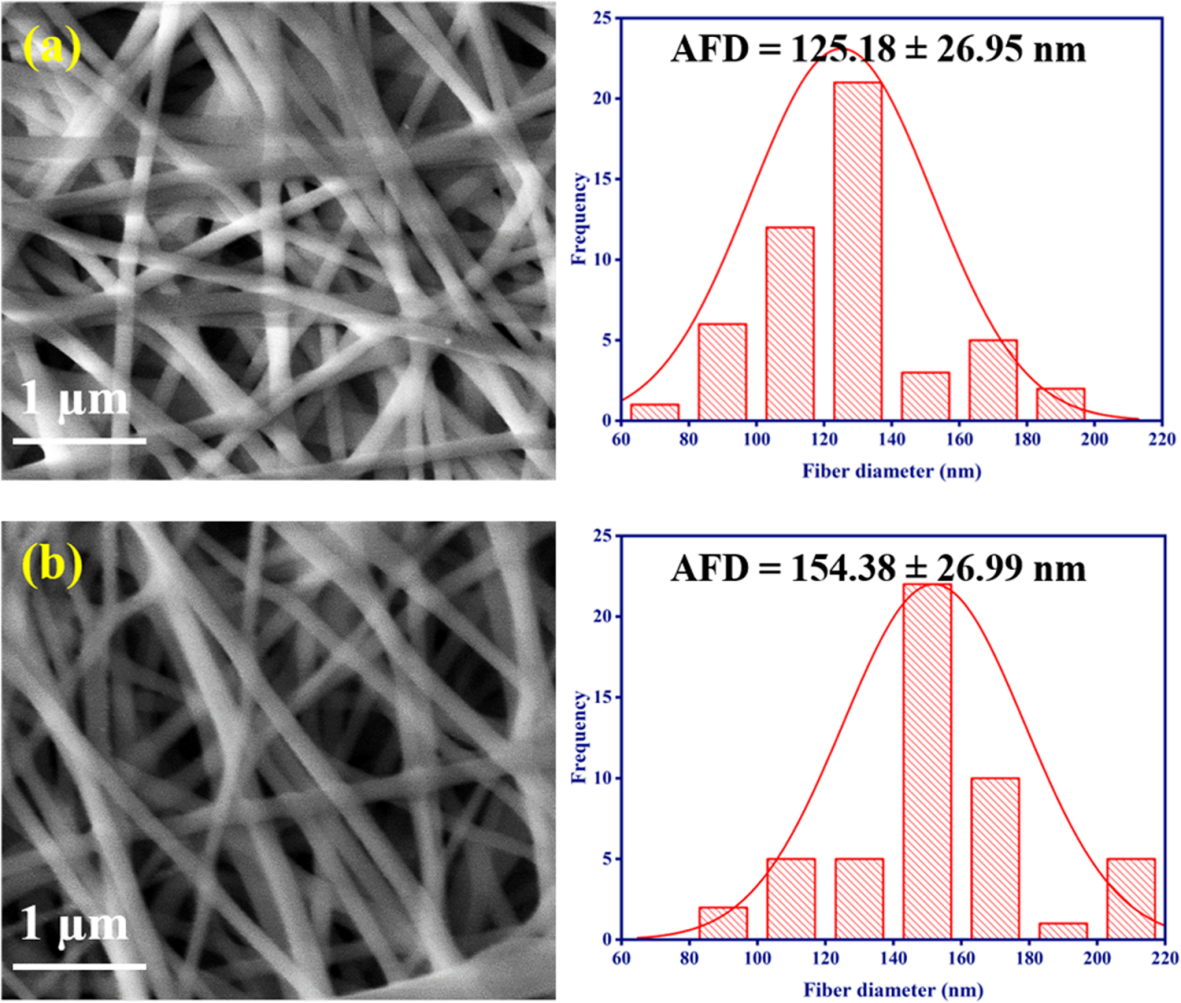
Morphology and diameter distributions of the nanofibrous scaffolds observed under scanning electron microscope: (a) PVA/HEC and (b) PVA/HEC-bromelain nanofibrous scaffolds.

Image processing software was used to calculate the average fiber diameter (AFD), which is approximately 125 nm for the PVA/HEC nanofibrous scaffold and 154 nm for the bromelain-PVA/HEC nanofibrous scaffold. Furthermore, no bromelain aggregates were seen on the surface of these bromelain-infused PVA/HEC nanofibrous scaffolds, indicating that bromelain molecules may be embedded within the fibrous matrix.

The bromelain-PVA/HEC nanofibrous scaffold was examined by HR-TEM analysis to confirm the inclusion of bromelain into the nanofibers and to more clearly expose the microstructure of the composite nanofibers. Figure 2 depicts the TEM images of the PVA/HEC and bromelain-PVA/HEC nanofibrous scaffolds.

**Figure 2:**
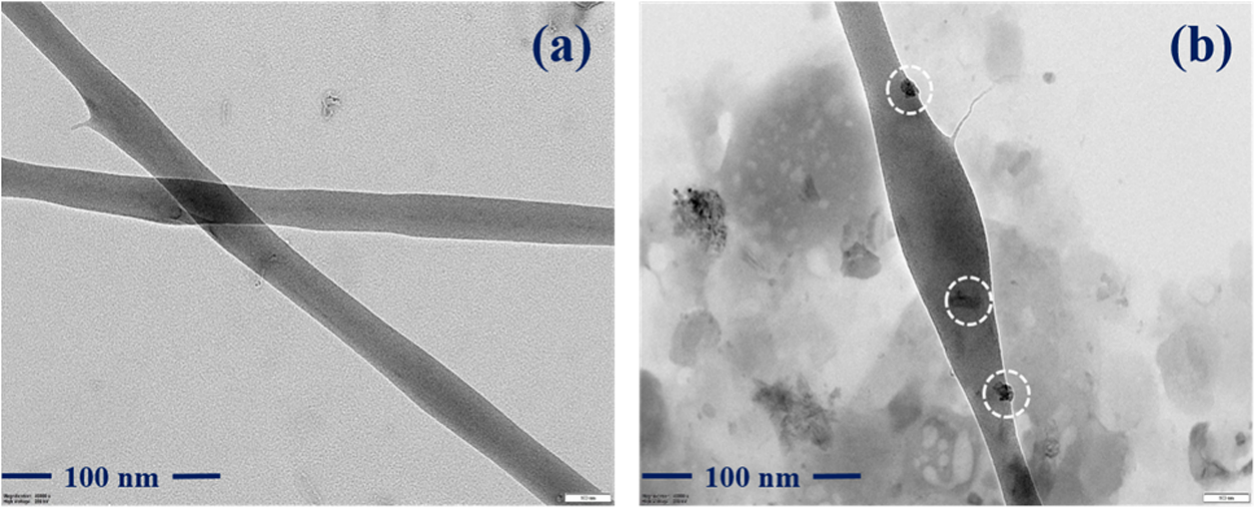
Transmission electron microscope images of the fabricated nanofibrous scaffolds: (a) PVA/HEC and (b) PVA/HEC-bromelain nanofibrous scaffolds.

However, the manufactured nanofibers have a smooth surface; as shown in Figure 2 (b) bromelain appears on the surface of the nanofibers and the successful decoration of bromelain on the fiber surface is visible.

The topography of pure PVA/HEC and bromelain-PVA/HEC nanofibrous scaffolds exhibits nanofiber morphology (Figure 3(a),(b)).

**Figure 3:**
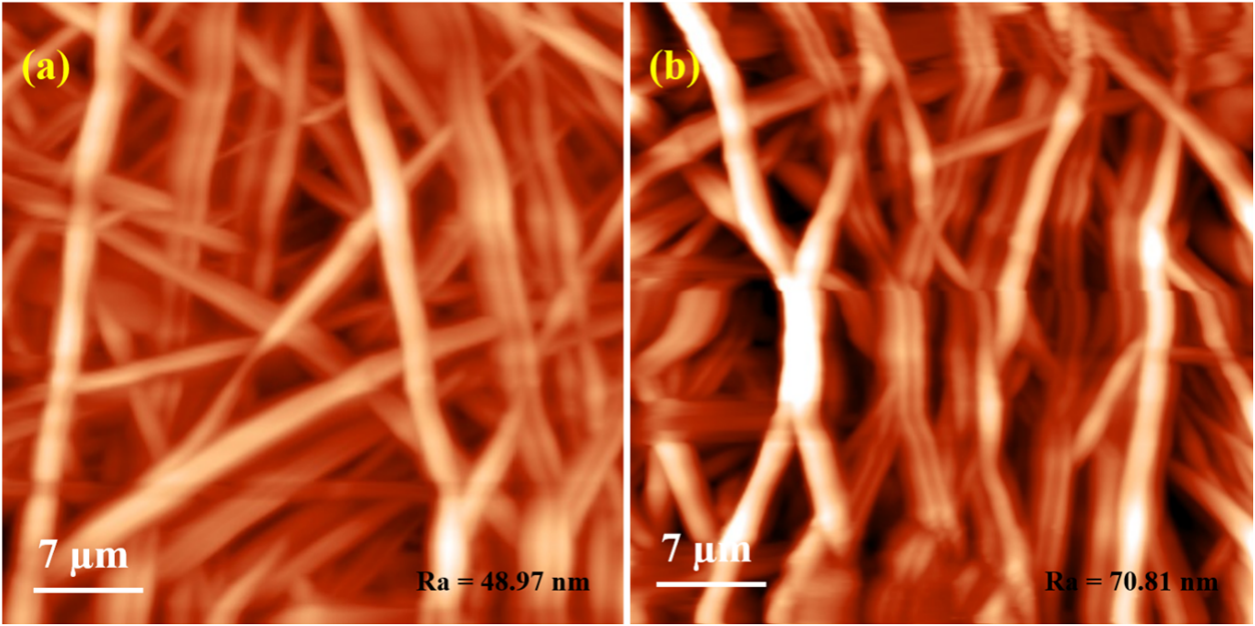
Atomic force microscope images of the nanofibrous scaffolds: (a) PVA/HEC and (b) PVA/HEC-bromelain nanofibrous scaffolds.

Individual fibers are visible and recognized on each AFM micrograph. The visual examination of the pristine PVA/HEC nanofibrous scaffolds reveals that the fibers are homogeneous and less densely packed. However, the bromelain-PVA/HEC nanofibrous scaffolds have densely packed fibers, which could be due to bromelain encapsulation on PVA/HEC nanofibrous scaffolds. The pristine PVA/HEC nanofibrous scaffolds had an average roughness of 48.97 nm over a 7 μm × 7 μm surface area. At the same surface area, the bromelain-PVA/HEC nanofibrous scaffolds had a much higher surface roughness of 70.81 nm, which is consistent with the fact that the fibers are tightly packed. AFM images revealed that the composite nanofibers exhibit higher porosity, making them suitable for various biomedical applications.^18^

### 3.2 Fourier-Transform Infrared Spectroscopy

ATR-FTIR spectroscopy was used to investigate the functional groups of the nanofibrous scaffolds and the interaction between them (Figure 4(A)).

**Figure 4:**
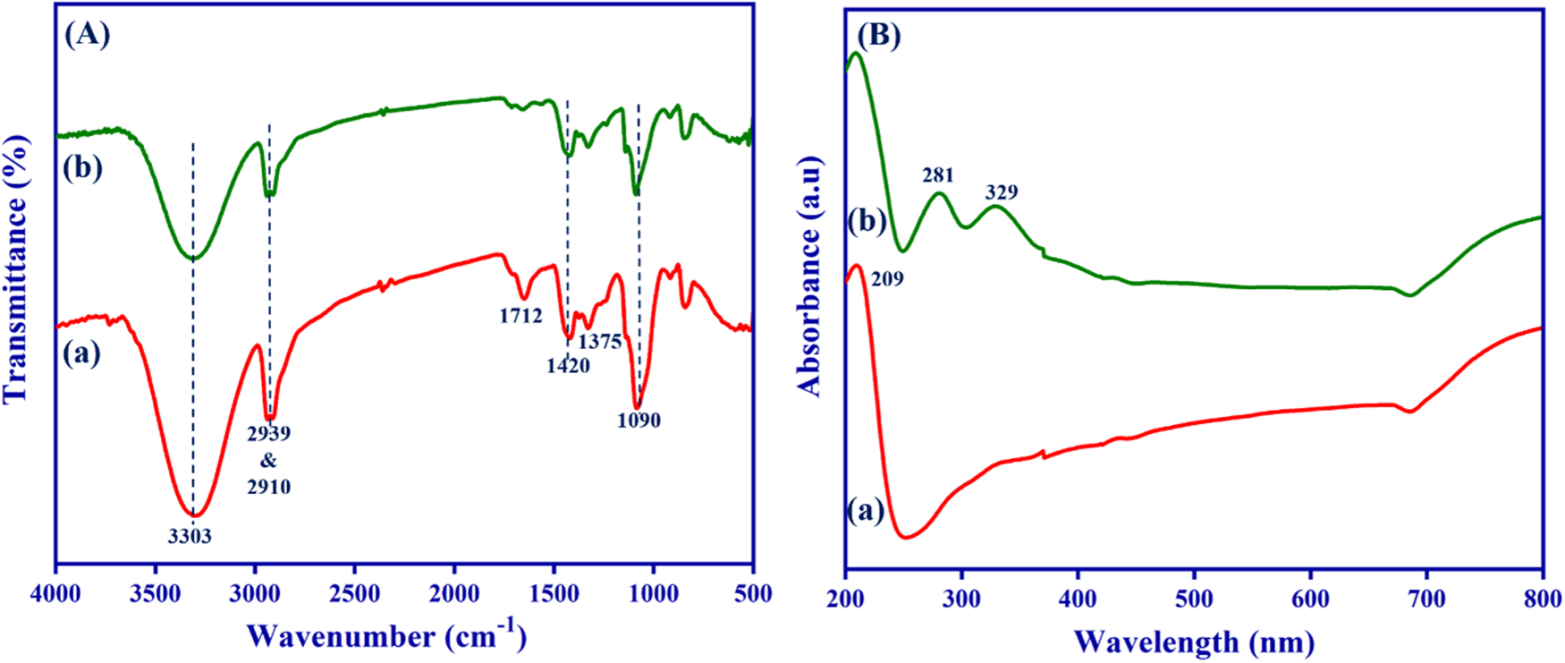
Comparative (A) FTIR spectra and (B) UV–vis-NIR absorbance spectra of the nanofibrous scaffolds: (a) PVA/HEC and (b) PVA/HEC-bromelain nanofibrous scaffolds.

When comparing the FTIR spectra of pure PVA and PVA/HEC nanofibers, the typical changes in apparent peaks confirmed the incorporation of HEC into the PVA matrix. These changes provide evidence of physical and possibly chemical interactions between the two polymers, PVA and HEC. The FTIR spectra of pure PVA and HEC are provided in Figure S1 (Supporting Information). The PVA/HEC nanofibrous scaffold showed an intense band at 3303 cm^–1^ corresponding to the stretching mode of the O–H group, followed by C–H stretching at 2939 cm^–1^ and 2910 cm^–1^.^40,41^ Meanwhile, the peak at 1712 cm^–1^ is associated with C=O stretching and is present in both PVA and HEC. The C–O–C stretching vibration appears at 1420 cm^–1^, while the C–OH in-plane stretching peak appears at 1375 cm^–1^. The C–O crystallinity peak of PVA was detected at 1090 cm^–1^. However, the peak seen in the 918–846 cm^–1^ region corresponds to C–H and CH_2_ bending vibrations.^42,43^ Remarkably, compared to the PVA/HEC nanofibrous scaffold, the intensity of the bromelain-infused nanofibrous scaffold was significantly reduced. This could be attributed to the strong intermolecular hydrogen bonding between the PVA/HEC nanofibrous scaffold, which confirmed the existence of the bromelain molecule.

### 3.3 Ultraviolet–Visible-Near Infrared Spectroscopy

To validate the existence of bromelain molecules, we measured their absorbance using UV–vis-NIR spectroscopy. The presence of the C=O and C=C groups of PVA was confirmed by the UV absorption peak of a typical PVA/HEC nanofibrous scaffold, which was at around 209 nm and validated the presence of both unsaturated ethylene groups and carbonyl groups in PVA,^44^ as illustrated in Figure 4 (B), (a). Bromelain exhibits a distinctive absorption peak in the range of 280–290 nm due to aromatic amino acid residues.^45^ After adding bromelain, the spectra of the composite nanofibrous scaffold displayed absorption maxima at 281 and 329 nm, respectively, due to the π-π* or n-π* electronic transition.^46^ This can be explained by the existence of bromelain in the PVA/HEC matrix, which was also proved by the FTIR measurements.

### 3.4 Differential Scanning Calorimetry Analysis

Figure 5(A) depicts DSC thermograms for electrospun PVA/HEC and bromelain-PVA/HEC nanofibrous scaffolds.

**Figure 5:**
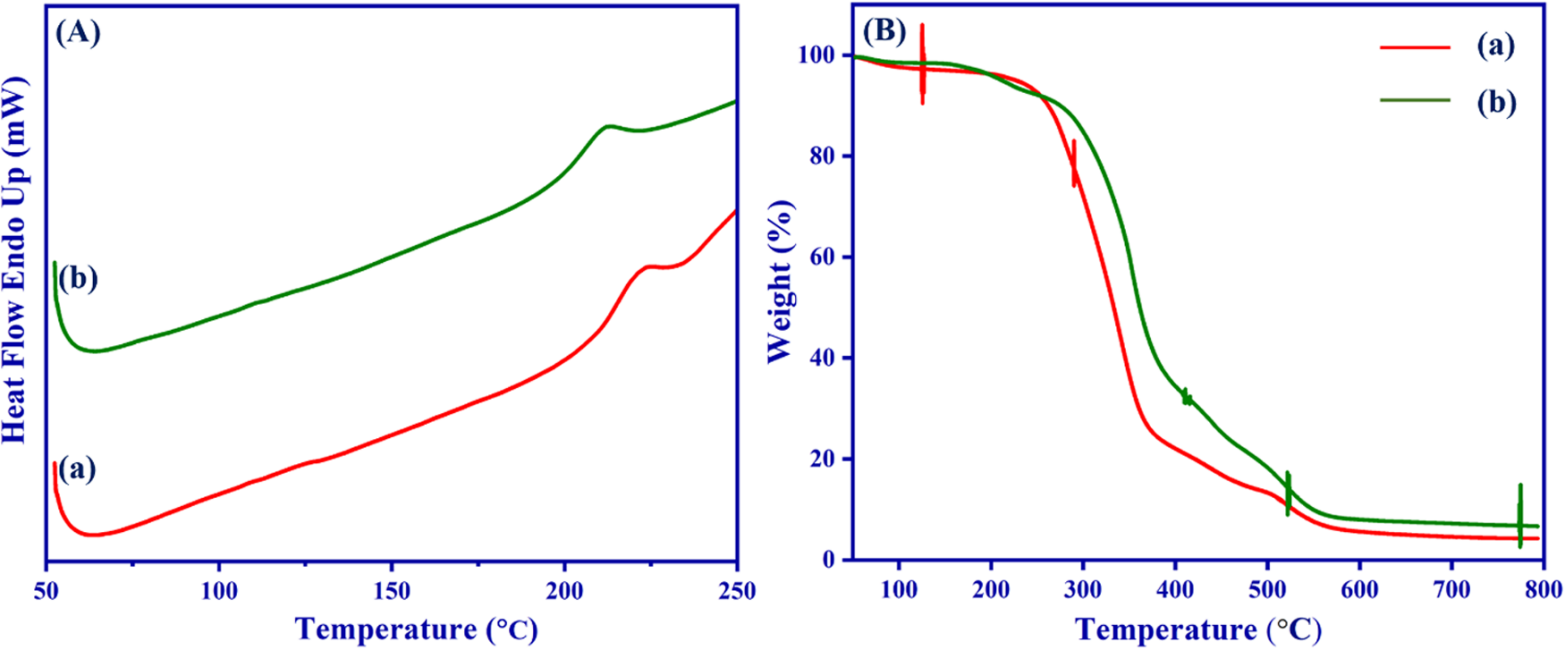
(A) DSC thermogram and (B) TGA analysis of the as-prepared nanofibrous scaffolds: (a) PVA/HEC and (b) PVA/HEC-bromelain nanofibrous scaffolds.

A very strong exothermic peak at around 220 °C [Figure 5(A), (a)] corresponds to the melting temperature of pure PVA and reflects the good miscibility between the two molecular chains, which is quite consistent with the given findings.^47,48^ This peak shifts to 211 °C when bromelain is added to PVA/HEC nanofiber; this gradually shifts the melting peak to lower temperatures as a result of the addition of bromelain molecules. This is due to the segmental motions of polymer chains during the electrospinning process, which were severely constricted by the strong interaction between hydrogen bonds.^49^

### 3.5 Thermogravimetric Analysis

The thermal stability of nanofibrous scaffolds can be analyzed using TGA at temperatures ranging from 50 to 800 °C. Figure 5(B) depicts the TGA thermograms for pristine PVA/HEC and bromelain-PVA/HEC nanofibrous scaffolds. According to the TGA data (Figure 5(B)), each sample showed three different phases of weight loss. Loss of moisture and physisorbed water molecules is associated with the first weight loss range of 53–235 °C.^50^ Besides, the decomposition of the PVA molecules’ side chains is responsible for the second one, which occurs between 235 and 370 °C, while the decomposition of the PVA main chain occurs between 370 and 520 °C.^51,52^ This clearly shows that the high HEC volume ratio in the PVA/HEC matrix and the encapsulation of bromelain in the nanofibrous scaffolds induce a large weight loss during the second stage of thermal decomposition.

### 3.6 Porosity

We assessed the porosity of bromelain-PVA/HEC nanofibrous scaffolds using an alcohol displacement technique. The porosity of the two different PVA/HEC nanofibrous scaffolds is displayed in Figure 6(A).

**Figure 6:**
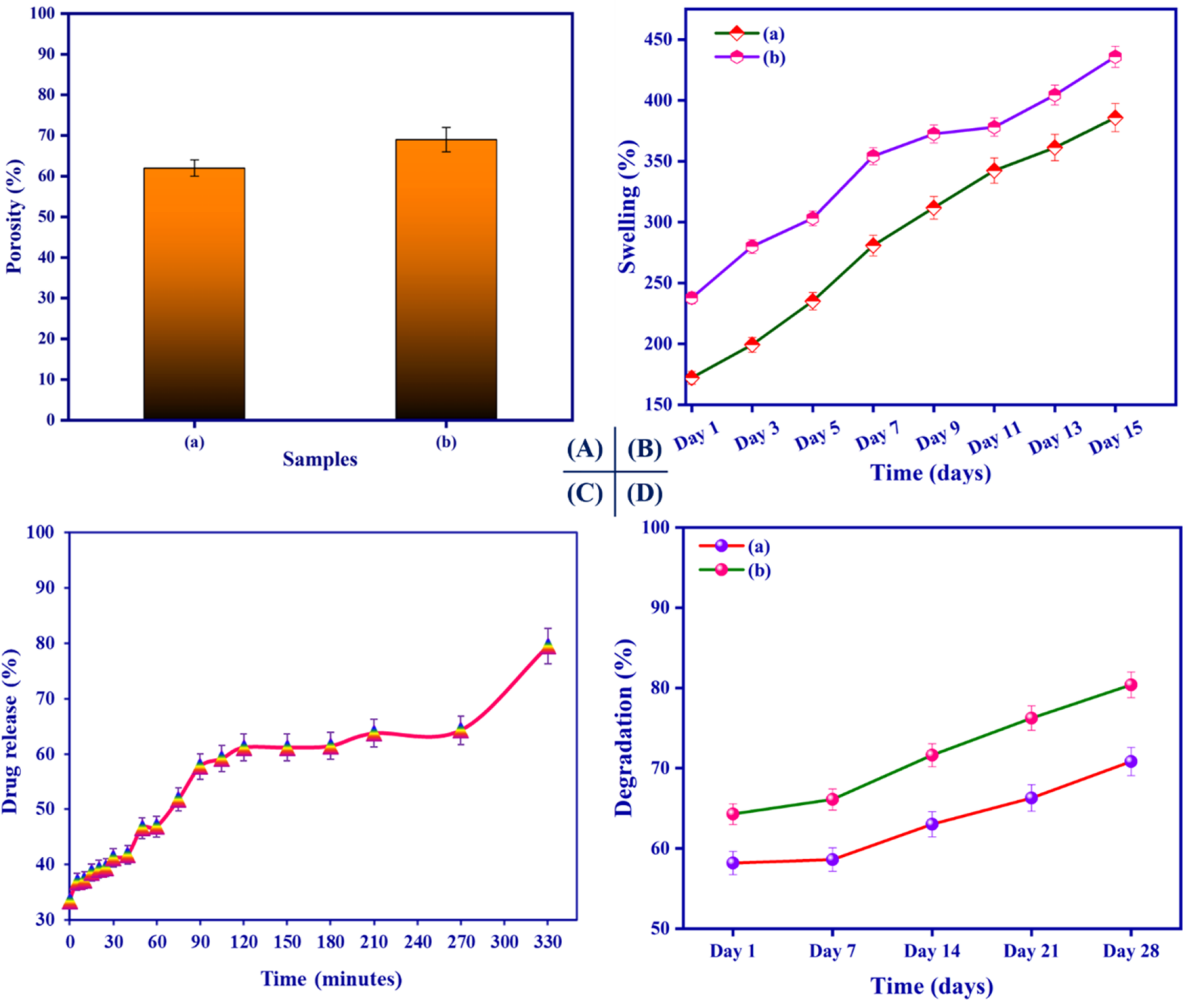
(A) Porosity analysis, (B) *in vitro* swelling capacity, (C) *in vitro* drug release profile, and (D) *in vitro* degradation studies of the nanofibrous scaffolds: (a) PVA/HEC and (b) PVA/HEC-bromelain nanofibrous scaffolds.

Both nanofibrous scaffolds had an optimal porosity ranging from 60 to 70%. Even after the addition of bromelain, there is no substantial difference in the porosity of the composite nanofibrous scaffold. It could be due to the increased AFD of the nanofibrous scaffold after the addition of bromelain. According to these findings, nanofibers with high porosity are suitable for cancer therapy.^53^

### 3.7 *In Vitro* Swelling Profile

The water-absorption capacity of nanofibers is one of the most important features of biomedical research. The swelling ratio of the prepared PVA/HEC nanofibrous scaffolds was studied, as illustrated in Figure 6(B). The PVA/HEC nanofibrous scaffold swelled up to 390% of its weight in PBS by day 15 (Figure 6(B), (a)). Meanwhile, the bromelain-PVA/HEC nanofibrous scaffold had a 440% swelling ratio at equilibrium, higher than the pure PVA/HEC nanofibrous scaffold’s swelling ratio. The integrated nanoporous structure of bromelain-PVA/HEC nanofibers is responsible for their prolonged moisture retention since they can slow down water evaporation effectively.^54,55^

### 3.8 *In Vitro* Drug Release Studies

The drug release profile of bromelain from the PVA/HEC nanofibrous scaffold was assessed and depicted in Figure 6(C). According to the release profile, the rate of release varied with concentration. As can be seen, the initial burst release of bromelain from the PVA/HEC nanofibrous scaffold lasted approximately 40 min. The initial release of bromelain from the PVA/HEC nanofibrous scaffold is due to swelling and desorption of the hydrophilic polymer matrix in the aqueous medium (PBS, pH ∼ 7.4). Furthermore, the release rate of bromelain increased with pore size, which can be explained by the fact that the highest bromelain content is encapsulated inside the nanofibrous scaffold and released only after the polymer has dissolved. As a result, the release of bromelain from the PVA/HEC nanofibrous scaffold produced a more suitable release rate and a prolonged release profile. This drug release property was favorable for inducing tumor cell proliferation by giving a suitable concentration of the anticancer agent throughout the therapy period.^56^

### 3.9 *In Vitro* Enzymatic Degradation Studies

The *in vitro* enzymatic degradation of the nanofibrous scaffolds was monitored and depicted in Figure 6(D). At all sampling intervals, the degradation rate of the PVA/HEC nanofibrous scaffold was much slower than that of the bromelain-PVA/HEC nanofibrous scaffold. In contrast, the composite nanofibrous scaffold showed a 70% degradation within 14 days of incubation. This large change appears to be owing to the effective interaction of bromelain with the PVA/HEC nanofibrous scaffold. Bromelain-PVA/HEC nanofibrous scaffolds degraded by 80% after 21 days of incubation in PBS solution, releasing PVA/HEC and bromelain into the media. As a result, the capacity of the bromelain-PVA/HEC nanofibrous scaffold to withstand degradation anticipates its potential as an effective biomaterial.

### 3.10 Antibacterial Activity

Bromelain has been regarded as the most extensively used antibacterial agent; it plays an important role in the breakdown of proteins, which are important components of bacterial membranes. The resulting nanofibrous scaffolds are more efficient against both gram-positive and gram-negative bacteria (Figure 7(A)).

**Figure 7:**
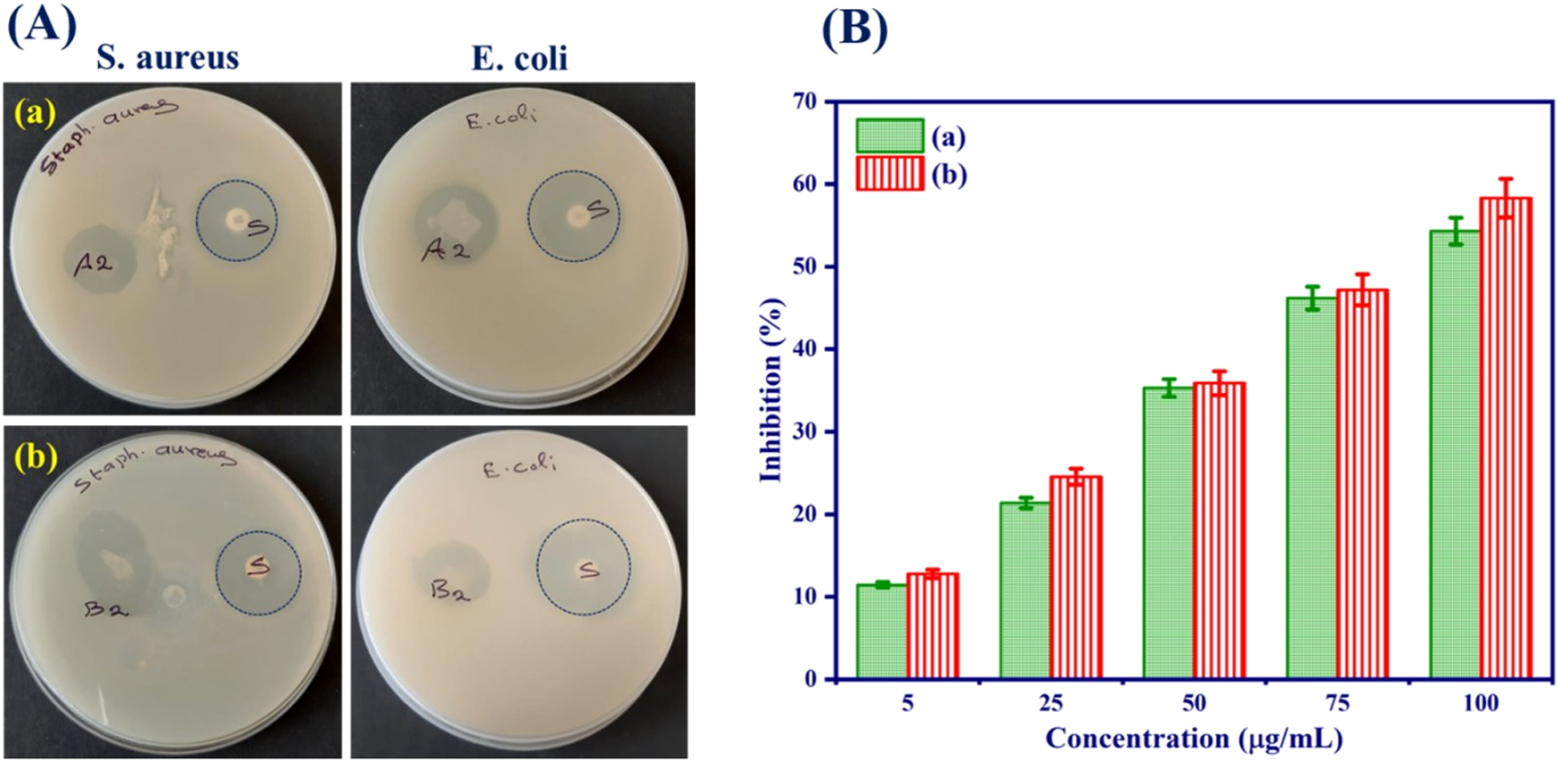
(A) *In vitro* antibacterial activity and (B) free radical scavenging activity of the nanofibrous scaffolds by DPPH assay: (a) PVA/HEC and (b) PVA/HEC-bromelain nanofibrous scaffolds.

The PVA/HEC nanofibrous scaffold inhibits bacterial growth. Figure 7(A) depicts the effect of bromelain on the adherence of *S. aureus* to the PVA/HEC nanofibrous scaffold. Bromelain’s mechanism for inhibiting bacterial growth is unknown. It has been proven that bromelain efficiently inhibits *S. aureus* cell proliferation (*P* < 0.05). As a result, the bromelain-PVA/HEC nanofibrous scaffold was more efficient against Gram-positive *S. aureus* (*P* < 0.05) than Gram-negative *E. coli* in terms of inhibition zone values (Table S1). This could be because bacteria have different cell wall structures and bromelain plays a significant role in this process by hydrolyzing peptide bonds found in the bacterial cell wall.^57^ The findings indicate that PVA/HEC nanofibrous scaffolds based on bromelain have great potential as eco-friendly antibacterial materials for a range of biomedical applications.

### 3.11 Antioxidant Activity

The protease enzyme bromelain has a high phenolic moiety, which allows it to scavenge free radicals. This 1,1-diphenyl-2-picrylhydrazyl (DPPH) assay was used to measure the antioxidant activity of the bromelain-PVA/HEC nanofibrous scaffold (Figure 7(B)). It has been found that the DPPH scavenging efficiency of the nanofibrous scaffolds depends on the concentration of bromelain in the PVA/HEC matrix. Radical scavenging efficiency increases with bromelain concentrations. As demonstrated in Figure 7(B), pristine PVA/HEC nanofibrous scaffolds have low antioxidant activity. Furthermore, the bromelain-PVA/HEC nanofibrous scaffold had a 58% antioxidant capacity (*P* < 0.05), demonstrating that the presence of polyphenolic chemicals in bromelain is responsible for its antioxidant activity.^58,59^ These nanofibrous scaffolds increased the antioxidant activity of the PVA/HEC polymer matrix, a significant finding for biomedical applications, especially for anticancer diagnostics.

### 3.12 *In Vitro* Cytotoxicity Assay

Nanofibrous scaffolds were tested for *in vitro* time-dependent cytotoxicity using the MTT assay to analyze the cell inhibition (Figure 8(A)–(C)).

**Figure 8:**
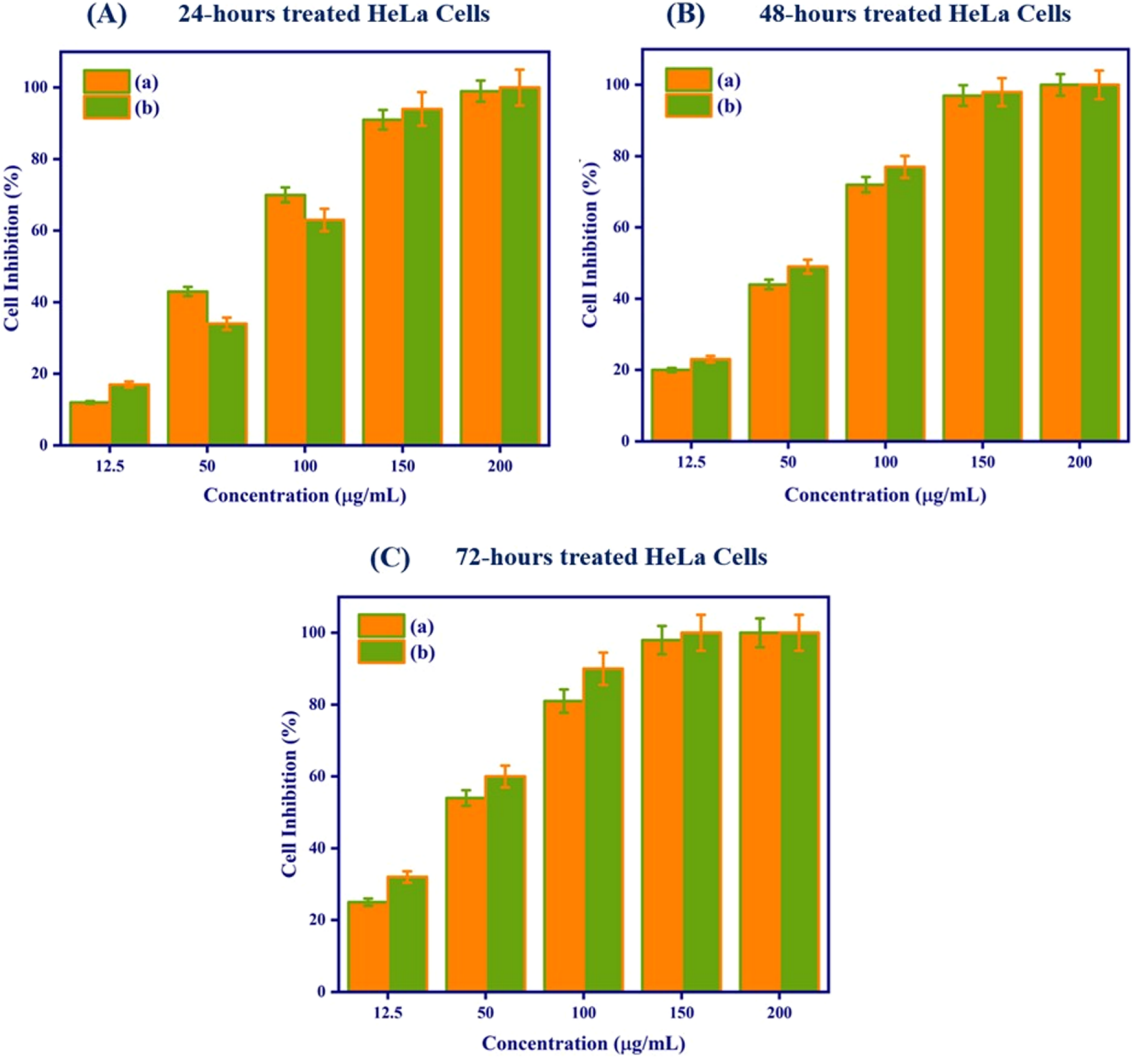
(A) MTT cell viability assay using HeLa cervical cancer cells after exposure to various concentrations and incubation times (24, 48, and 72 h). (a) PVA/HEC and (b) PVA/HEC-bromelain nanofibrous scaffolds.

The human cervical cancer HeLa cell lines were treated with 12.5, 50, 100, 150, and 200 μg/mL of the prepared nanofibrous scaffolds and incubated for 24, 48, and 72 h. Microscopic images of HeLa cells after 24, 48, and 72 h incubation times of the prepared nanofibrous scaffolds are given (Figure S3, Supporting Information). In the HeLa cell line, PVA/HEC nanofibrous scaffolds inhibited cell proliferation more than bromelain-PVA/HEC nanofibrous scaffolds across a 24-h incubation period at concentrations up to 100 μg/mL. In this study, PVA/HEC nanofibrous scaffolds inhibited HeLa cells by 12, 43, 70, 91, and 99%, and bromelain-PVA/HEC nanofibrous scaffolds showed 17, 34, 63, 94, and 100% cell inhibition after 24 h. As can be seen in Figure 8 (A), at concentrations of 50 and 100 μg/mL, PVA/HEC nanofibrous scaffolds inhibit cells more effectively than bromelain-PVA/HEC nanofibrous scaffolds. This is due to the inherent characteristics or functional groups found in the PVA/HEC matrix, which interact more effectively with cell components, resulting in increased cell inhibition. At higher concentrations (150 and 200 μg/mL) or longer exposure times (48 and 72 h), the enzymatic activity of bromelain disrupts typical cellular structures and enhances its cytotoxicity. These results showed that bromelain-PVA/HEC nanofibrous scaffolds had higher anticancer efficacy (*P* < 0.05) than pristine PVA/HEC nanofibrous scaffolds with longer incubation times and concentrations, as shown in Figure 8. This is most likely caused by the concentration of bromelain and the direct interaction between cells and nanofibrous scaffolds. Bromelain, a proteolytic enzyme, is known for its anticancer effects, such as triggering apoptosis, regulating the immune response, and limiting metastasis. Adding bromelain could provide a bioactive function that the PVA/HEC blend alone cannot provide, such as targeted anticancer activity or improved biocompatibility. A study by Bhui et al. reported that bromelain inhibits cancer cell growth in a concentration and time-dependent manner. Furthermore, half-maximal inhibitory concentration (IC_50_) values were determined using time-dependent graphs obtained after treating HeLa cells with PVA/HEC and bromelain-PVA/HEC nanofibrous scaffolds. Analysis showed that bromelain-PVA/HEC nanofibrous scaffolds resulted in lower IC_50_ values (39.2 μg/mL) than pristine PVA/HEC nanofibrous scaffolds (44.56 μg/mL) at 72 h′ incubation time. This suggested that PVA/HEC nanofibrous scaffolds successfully achieved the delivery of bromelain to the targeted cells. These outcomes concur with those of earlier research that assessed bromelain’s anticancer properties on various cancer cell types *in vitro*.^60^ According to our research, bromelain may have some antioxidant properties and be useful in lowering oxidative stress and preventing cancer.^34^

## 4 Conclusions

In summary, the fabrication and *in vitro* bioactivity of nanofibrous scaffolds were extensively investigated. The *in vitro* bioactivity of nanofibrous scaffolds is significantly influenced by their surface morphology and specific surface area. The incorporation of bromelain on PVA/HEC nanofibers not only improves the porosity and degradation properties but also provides high bromelain loading capacities, allowing the enzyme to be released in a sustained and prolonged manner, resulting in higher *in vitro* antitumor efficacy than pristine nanofibrous scaffolds. These results demonstrated the great therapeutic potential of bromelain-loaded PVA/HEC nanofibrous scaffolds, which may aid in the personalization of cancer theranostics.

## Supplementary Material



## Data Availability

The data underlying this study are available in the published article and its Supporting Information.

## Notes

FTIR spectra of pristine PVA and HEC (Figure S1); XRD analysis of PVA, HEC, and PVA/HEC nanofibrous scaffold (Figure S2); microscopic images of HeLa cells after 24, 48, and 72 h incubation times of the prepared nanofibrous scaffolds (Figure S3); inhibition zone results were taken after 24 h of incubation at 37 °C against *S. aureus* and *E. coli* bacterial strains for the prepared nanofibrous scaffolds (Table S1) (PDF)
