# Supplementary material for: Bromelain-Infused Poly(vinyl alcohol)/Hydroxyethyl Cellulose Nanofibrous Scaffolds for Cancer Therapy: Fabrication, Characterization, and In Vitro Assessment
Source: ACS Omega. 2025 May 15;10(20):20106–16. doi: 10.1021/acsomega.4c09347 (PMC12120600; doi:10.1021/acsomega.4c09347)
Supplement: Supplementary file 1 [file ao4c09347_si_001.pdf]

Supporting information

Bromelain-infused Polyvinyl alcohol/Hydroxyethyl cellulose Nanofibrous Scaffolds for Cancer Therapy: Fabrication, Characterization and *In vitro* Assessment

Suganya Bharathi Balakrishnan[a], Lilian Ibrahim[b], Esakkimuthu Shanmugasundaram[c], Na'il Saleh[b*], and Stalin Thambusamy*[c]

[a] Dayananda Sagar University, Department of Chemistry, School of Engineering, Devarakaggalahalli, Harohalli, Ramanagara, Karnataka, India.

E-mail: sbsuganyaa@gmail.com and suganyabharathi-chem@dsu.edu.in

[b]Department of Chemistry, College of Science, United Arab Emirates University, P.O. Box 15551, Al Ain, United Arab Emirates.

[c] Department of Industrial Chemistry, School of Chemical Sciences, Alagappa University Karaikudi, Tamil Nadu, India.

*Corresponding author e-mail: ***Stalin Thambusamy -** *stalin.t@alagappauniversity.ac.in.

***Na'il Saleh -** n.saleh@uaeu.ac.ae.

Fourier transform infrared spectroscopy

In pure PVA, the O-H stretching band appears at 3292 cm^{-1}, and after the incorporation of HEC, shifts in the O-H band to a higher wavenumber 3303 cm^{-1} due to hydrogen bonding interaction between the PVA and HEC indicating increased intermolecular hydrogen bonding.

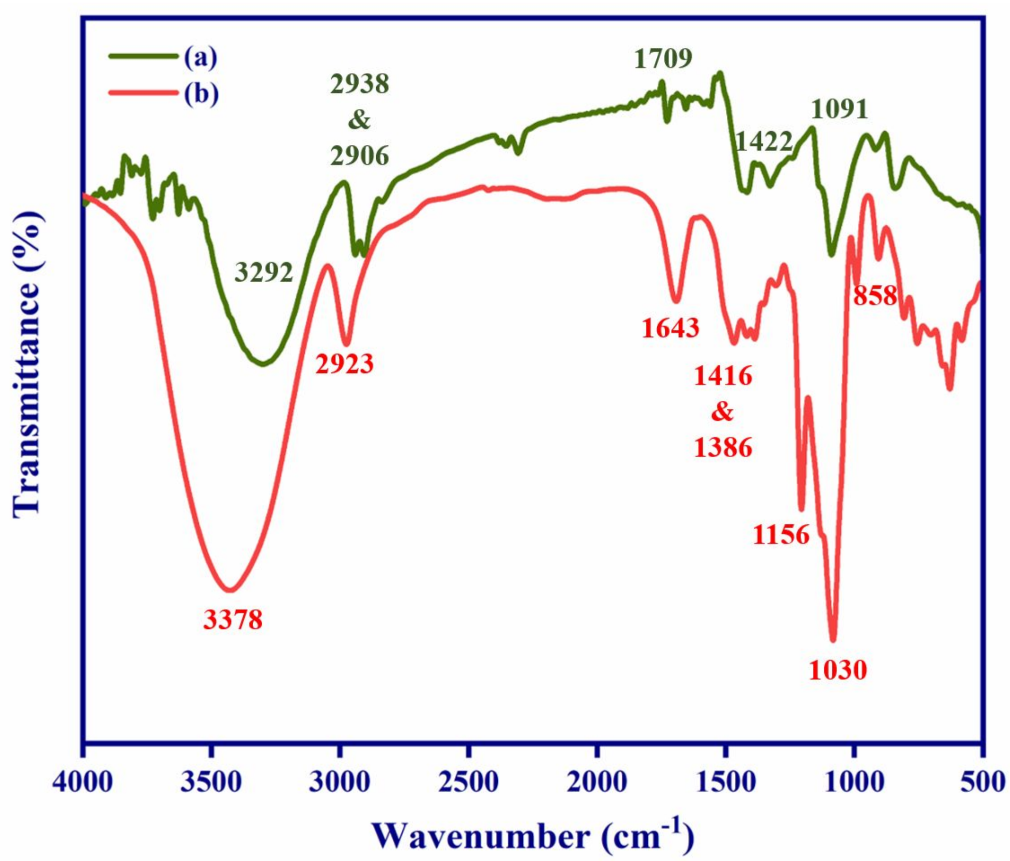

Figure S1. FTIR spectra of (a) pristine PVA and (b) HEC.

These changes provide evidence of physical and possibly chemical interactions between the two polymers PVA and HEC. This can be seen in Figure S1, shows the FTIR spectra for pure PVA and HEC. Compared to pristine PVA (2938 & 2906 cm^{-1}) slight change was observed in PVA/HEC nanofibers (2939 & 2910 cm^{-1}) in the symmetric & asymmetric stretching of the alkyl group and changes in peak intensity reflected the modifications in the vibrational environment caused by HEC incorporation. Alterations in the C=O stretching band of PVA/HEC nanofibers

(1712 cm^{-1}) in contrast to PVA (1709 cm^{-1}) might indicate the interaction between PVA's hydroxyl groups and HEC's ether or hydroxyl groups, possibly through hydrogen bonding [1]. The peak at 1422 cm^{-1} in pristine PVA is shifted to 1420 cm^{-1} in PVA/HEC nanofibers suggesting the overlapping of PVA and HEC functional groups in the PVA/HEC nanofibers can signify the incorporation of HEC in the PVA polymer matrix. The observed FTIR spectrum changes provide strong evidence that HEC was incorporated into the PVA matrix, most likely by molecular hydrogen bonding.

X-ray diffraction analysis

Polyvinyl alcohol (PVA) and hydroxyethyl cellulose (HEC) have unique X-ray diffraction (XRD) patterns due to their molecular structures and crystallinity. PVA is semi-crystalline. Its XRD pattern contains a peak due to the organized arrangement of polymer chains in crystalline areas. PVA's typical diffraction peak at around $2\theta = 19.1°$ observed in the XRD pattern in Figure S2. HEC is typically amorphous because it replaces hydroxyl groups with hydroxyethyl groups, which prevents the formation of crystalline structures. Its XRD pattern would likely exhibit broad humps rather than sharp peaks, indicating an amorphous nature. The broad peak for cellulose derivatives, such as HEC, appeared at $2\theta = 20.6$ ° (Figure S2). PVA will show sharper and more intense peaks, indicating crystalline zones, whereas HEC peaks will be broad and diffuse, indicating an amorphous polymer [2]. Besides, PVA/HEC nanofibrous scaffolds the shift in the diffraction peak from 19.1° to 19.6° indicates strong interactions between PVA and HEC, a clear indication of the structural changes in the PVA/HEC nanofibers due to the incorporation of HEC into PVA.

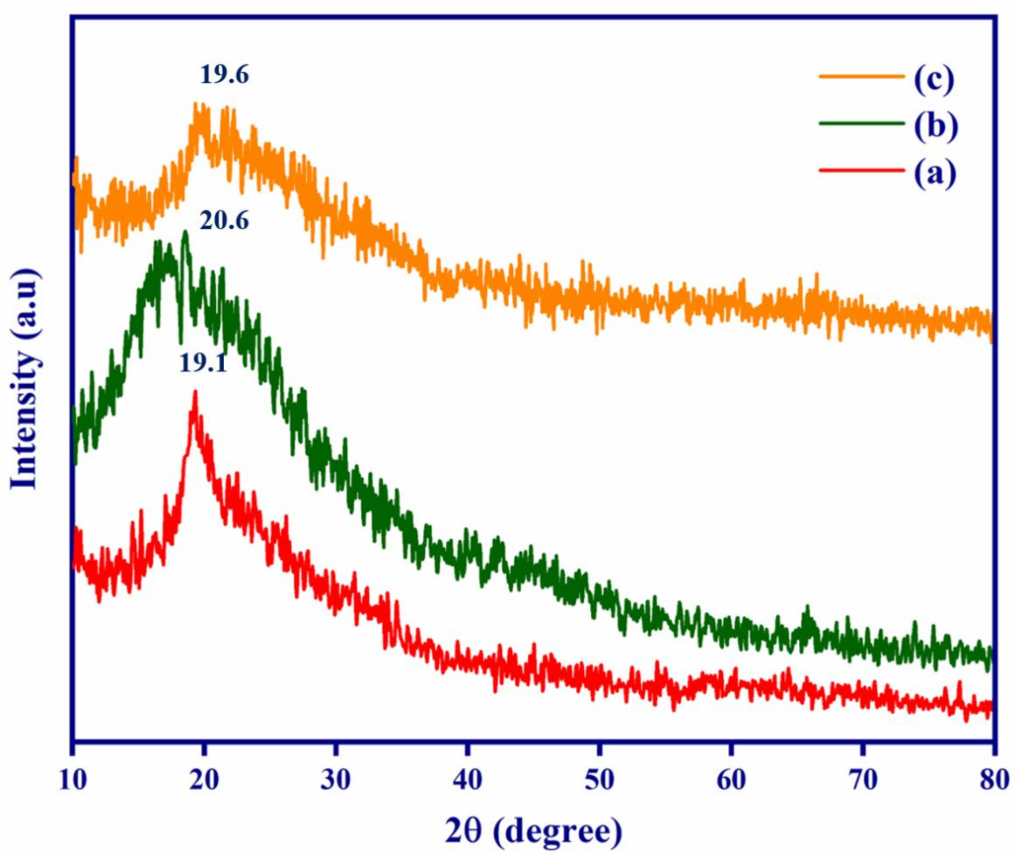

Figure S2. XRD analysis of (a) pristine PVA (b) HEC and (c) PVA/HEC nanofibrous scaffold.

Table S1. Inhibition zone results were taken after 24 hours of incubation at 37 °C against *S. aureus* and *E.coli* bacterial strains for the prepared nanofibrous scaffolds.

**Samples (Nanofibrous scaffolds)**	**Zone of inhibition (mm)**	
	S. aureus	***E. coli***
**PVA/HEC**	**13.59 ± 0.75**	**14.41 ± 0.58**
**Bromelain-PVA/HEC**	**15.27 ± 1.12**	**14.14 ± 0.94**

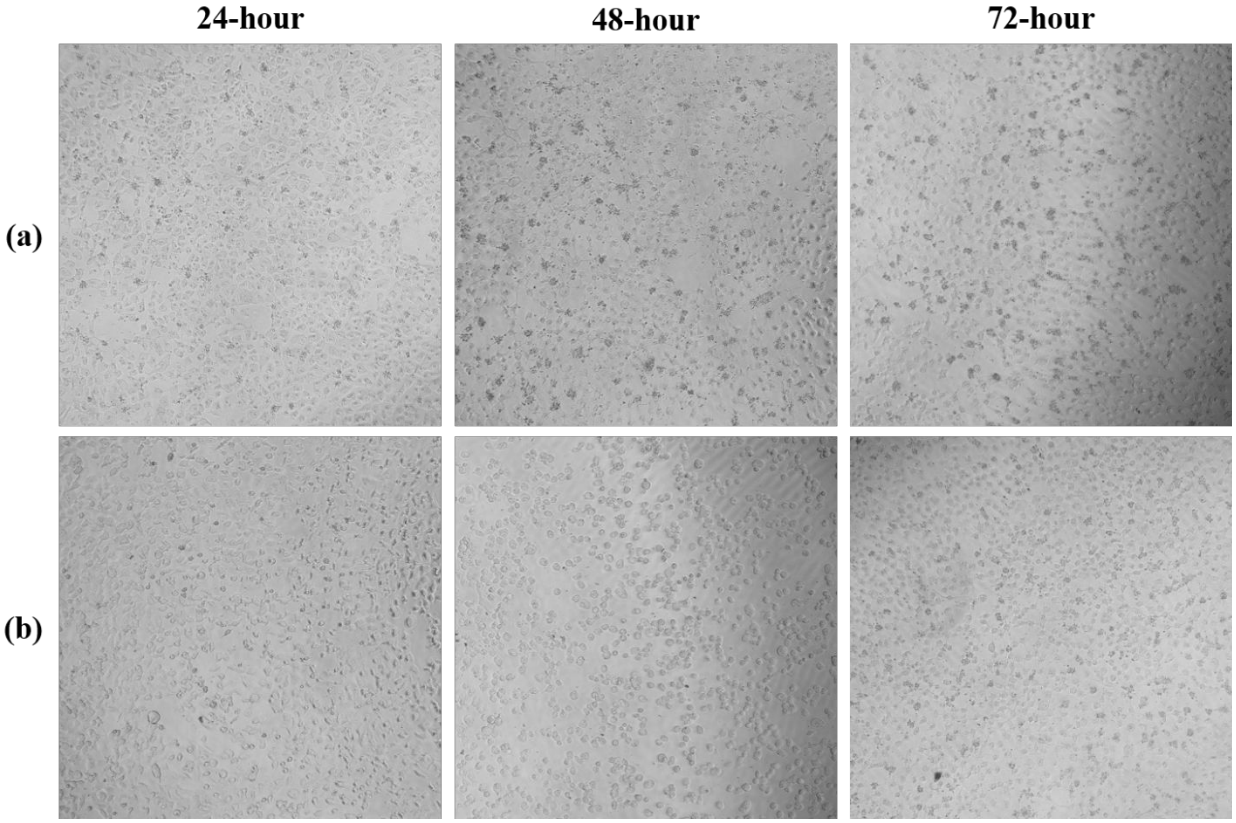

Figure S3. Microscopic images of HeLa cells after 24, 48, and 72 h incubation times. (a) PVA/HEC and (b) PVA/HEC-bromelain nanofibrous scaffolds.

References

[1] Liu, F.; Goodarzi, A.; Wang, H.; Stasiak, J.; Sun, J.; Zhou, Y.; Chahal, S.; Fathima, S.J.H.; Yusoff, M.B.M. Biomimetic growth of bone-like apatite via simulated body fluid on hydroxyethyl cellulose/polyvinyl alcohol electrospun nanofibers. *Biomed. Mater. Eng.* **2014**, *24*, 799-806.

[2] El Fawal, G.; Hong, H.; Song, X.; Wu, J.; Sun, M.; Zhang, L.; He, C.; Mo, X.; Wang, H. Polyvinyl alcohol/hydroxyethylcellulose containing ethosomes as a scaffold for transdermal drug delivery applications. *Appl. Biochem. Biotechnol.* **2020**, *191*, 1624-1637.
